# High Quality Factor in Solution-Processed Inorganic Microcavities Embedding CsPbBr_3_ Perovskite Nanocrystals

**DOI:** 10.1021/acsaom.3c00157

**Published:** 2023-07-18

**Authors:** Simone Bertucci, Andrea Escher, Matilde Cirignano, Manuela De Franco, Federico Locardi, Maddalena Patrini, Davide Comoretto, Paola Lova, Francesco Di Stasio

**Affiliations:** † Photonic Nanomaterials, Istituto Italiano di Tecnologia, Via Morego 30, 16163 Genova, Italy; ‡ Dipartimento di Chimica e Chimica Industriale, Università degli Studi di Genova, Via Dodecaneso 31, 16146 Genova, Italy; § Dipartimento di Fisica, Università degli Studi di Pavia, Via Agostino Bassi 6, 27100 Pavia, Italy

**Keywords:** photonic crystals, optical microcavity, solution processing, perovskite nanocrystals, light management

## Abstract

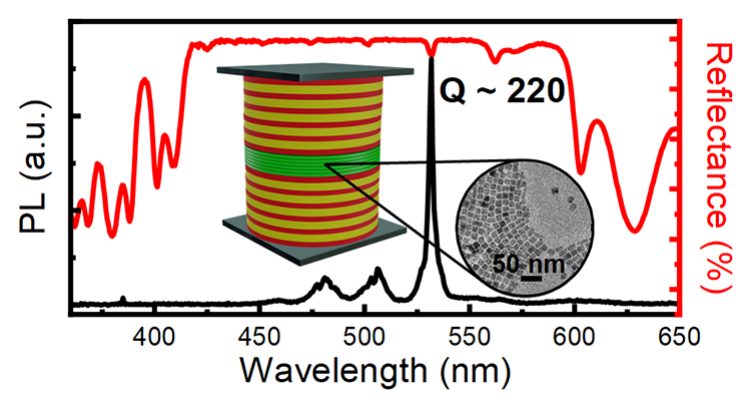

Optical microcavities grant manipulation over light–matter interactions and light propagation, enabling the fabrication of foundational optical and optoelectronic components. However, the materials used for high-performing systems, mostly bulk inorganics, are typically costly, and their processing is hardly scalable. In this work, we present an alternative way to fabricate planar optical resonators via solely solution processing while approaching the performances of conventional systems. Here, we couple fully solution-processed high dielectric contrast inorganic Bragg mirrors obtained by sol–gel deposition with the remarkable photoluminescence properties of CsPbBr_3_ perovskite nanocrystals. The approach yields microcavities with a quality factor of ∼220, which is a record value for solution-processed inorganic structures, and a strong emission redistribution resulting in a 3-fold directional intensity enhancement.

## Introduction

One of the greatest challenges facing the scientific community today is the ability to manipulate light. Full control over the properties of light would benefit many important technological applications, ranging from photovoltaics,^1,2^ photocatalysis,^3,4^ telecommunications,^5,6^ and modulators.^7−9^ planar photonic crystal microcavities (MCs) made of distributed Bragg reflectors (DBRs)^10,11^ are well known to modulate the dielectric environment of an emitter embedded in the defect layer. Such a modulation can indeed affect its emission spectrum by redistributing the strength of the fluorescence oscillator. This capability, as well as the spectral features of a microcavity and its finesse, is strongly related to the dielectric contrast between the media in the DBR.^12−14^ Conventional MCs are indeed made of inorganic materials with a high refractive index (*e.g*., Si, GaN, ZrO_2_, TiO_2_)^15−17^ coupled with air voids or SiO_2_^18^ deposited via high-vacuum techniques.^19−21^ These methods, despite having the best performance and control over thickness and roughness of thin films, are difficult to scale and costly, especially from an energetic point of view. In recent years, various alternative materials processed from solution have been explored to mitigate fabrication costs. Among them, commodity,^22,23^ specifically tailored technical polymers,^24−27^ and nanocomposites^28,29^ have greatly improved the performance of available systems, reaching a maximum value of Δ*n* = 0.35,^30^ but are still far from achieving the dielectric contrast typical of inorganics. In addition, polymer–inorganic hybrid materials have recently attracted particular attention due to their versatility and superior performance compared to pristine polymers.^31−33^ To this end, hybrid materials that exhibit refractive index values comparable to state-of-the-art metal-oxides, while retaining the ease of manufacturing of solution-processed polymer structures,^34^ can be prepared by sol–gel reaction of organic titania and silica precursors in the presence of a polymeric stabilizer responsible for film formation and stabilization during spin-coating deposition. Such films are then annealed at relatively low temperature (80–300 °C), allowing the tuning of porosity and refractive index while maintaining subnanometric roughness and transparency.^34^

In this work, we use an improved version of similar materials to fabricate solution-processed DBRs, which are then employed to build an optical microcavity embedding a green-emitting CsPbBr_3_ perovskite colloidal nanocrystal (PNC) film. Among metal halides, CsPbBr_3_-based ones stand out for their performance^35−38^ and mild synthesis conditions^39^ but their poor stability against environmental stressors hindered their practical application.^40^ Compatibility issues can be alleviated by employing such materials in systems fabricated entirely under mild conditions. We evaluated the light confinement capabilities of our materials by optically characterizing this solution-fabricated system, highlighting the enhancement effect of the photonic environment on the photoluminescence of CsPbBr_3_ nanocrystals (NCs).

## Results and Discussion

Thin films of titania and silica are prepared via spin coating according to the procedure described in the Experimental Methods section and to the schematic of the deposition process shown in Figure S1. Briefly, the samples are spun-cast starting from butanol sols stabilized with poly(acrylic acid) of titanium (IV) butoxide and tetraethyl orthosilicate for titania and silica, respectively. By controlling the concentration of reagents in the sol, the amount of solution cast, and the rotation speed during deposition, we modulate the film thickness, providing a fundamental tool to tune the photonic band gap (PBG) of the structure to different spectral ranges. After deposition, thermal annealing on a standard laboratory hot plate at 500 °C for titania and 300 °C for silica films ensures the formation of the oxides. The temperature was chosen to ensure the best compromise between film quality, in terms of optical response homogeneity and transparency, and achievable refractive index values. For example, the silica precursor, which contains a large amount of polymer stabilizer, allows for a higher degree of porosity upon thermal degradation, thus decreasing the refractive index. Indeed, annealing at temperatures above 300 °C could cause the structure to collapse and affect transparency. Therefore, the material is first annealed at 300 °C and then at 500 °C after the deposition of the subsequent titania film.

**Figure 1:**
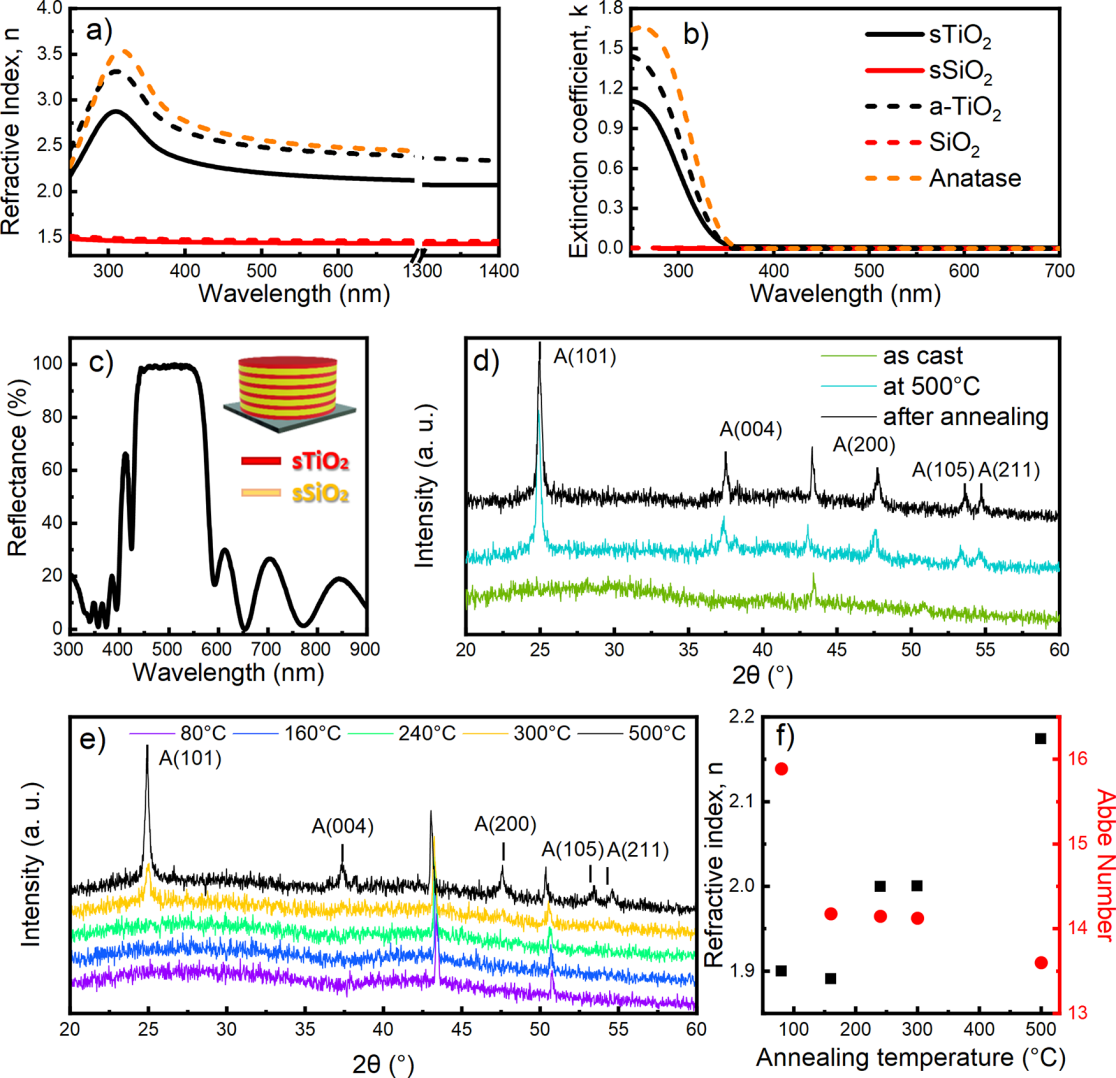
(a, b) Refractive index and extinction coefficient values for solution-processed titania (sTiO_2_, black line) and silica (sSiO_2_, red) and for thin films from high-vacuum techniques from the literature (amorphous TiO_2_ in a dashed black line,^41^ SiO_2_ in a dashed red line,^42^ anatase TiO_2_ in orange^41^); (c) reflectance spectrum for a 5.5 bilayer silica:titania DBR; (d) X-ray diffraction (XRD) pattern of sTiO_2_ thin film acquired as cast (green line), at 500 °C (cyan line) and at room temperature after cooling the sample (black line); (e) XRD patterns for different temperatures of annealing; and (f) refractive index values collected at 550 nm and calculated Abbe numbers as a function of annealing temperature.

Since the dielectric contrast between materials employed plays a key role in defining the optical response of the system, we first determined the refractive index dispersion of our media. The optical function of silica (sSiO_2_) and titania (sTiO_2_) thin films, retrieved by spectroscopic ellipsometry measurements, and their counterparts produced via high-vacuum techniques are shown in Figure 1a,b (the latter are taken from the literature).^47−49^ For the sTiO_2_ film (black continuous line), the refractive index increases from 2.02 in the near-infrared to 2.37 at 380 nm. The value further increases up to 310 nm where it reaches its maximum (2.9). As expected, the material is highly dispersive and has an Abbe number of 13.6, which falls in between literature data for amorphous (14.5) and anatase (12.6) TiO_2_ phases,^41^ in agreement with a semicrystalline composition (see Supporting Information eq 1). The extinction coefficient dispersion reported in Figure 1b shows that the material is transparent up to 350 nm, where strong UV absorption increases. Regarding silica, the material is less dispersive (Abbe number ∼75) and its index ranges from 1.44 at 1400 nm to 1.47 at 300 nm while it is fully transparent in the investigated range as the extinction coefficient approaches zero. The values retrieved for our film are smaller than those of material obtained using high-vacuum deposition techniques (dashed lines in Figure 1a). Such a difference is attributed to the presence of porosity typical of sol–gel materials and observed in a previous work.^34^ The extinction coefficients (Figure 1b) are negligible in the visible range for both materials, while we observe the offset of the TiO_2_ absorption below 350 nm, in agreement with the literature.^34,43^ The data confirm the high transparency and optical quality of the films.

Coupling titania and silica in subsequent depositions allows for the growth of tunable and highly reflective DBRs. A typical optical response for an 11 layers DBR (5.5 bilayers) is reported in Figure 1c together with the structure schematics. The spectrum shows a band with a 100% reflectance ranging from the violet to the orange parts of the visible spectrum, assigned to the PBG. Such a broad PBG arises thanks to the high dielectric contrast achieved between the two materials, which approaches Δ*n* = 0.73 at 550 nm, and currently represents the highest value reported in the literature for solution-processed DBRs and MCs.^17,18^ Such a large dielectric contrast owes to the low silica refractive index (1.44 at 550 nm) caused by the material porosity^34^ and to the high refractive index obtained for the titania (2.17 at 550 nm), thanks to the relatively high crystallinity of the film annealed at 500 °C. Figure 1d displays the X-ray diffraction (XRD) pattern collected for a sTiO_2_ film as cast (green line), during the annealing at 500 °C (cyan line) and after the annealing once the sample temperature reaches room condition (black line), thus reproducing the deposition and annealing process. In the film as cast, no specific diffraction peaks can be detected due to the amorphous nature of the material, while we can clearly observe peaks located at 2θ = 24.9, 37, 48, 54, and 55° upon annealing at 500 °C, which are assigned to the nanocrystalline anatase phase due to the characteristic broadened signals in agreement with the literature.^44^ After cooling, the peaks slightly shift to larger angles due to the modest thermal crystal contraction. The peak observed at 2θ ∼ 43° in all of the patterns is attributed to the stainless steel sample holder. To further evaluate the effect of crystallinity on the refractive index of sTiO_2_ thin films, we compared the XRD patterns collected upon annealing at different temperatures in Figure 1e. In XRD patterns acquired on films treated at 80, 160, and 240 °C, we do not observe specific peaks attributable to the sample but only signals ascribable to the sample holder at 2θ ∼ 43° and 51°. However, starting at 300 °C, we can clearly identify a weak peak at 24.9° typical of the anatase phase,^44^ which indicates the onset of the crystallization process. Further increasing the treating temperature to 500 °C causes the peak at 2θ = 24.9° to drastically increase in intensity and the formation of the diffraction signals previously described.^44^ Focusing now on refractive index values at 550 nm obtainable for the different annealing temperatures (Figure 1f), values reported for 80 and 160 °C do not differ significantly, in agreement with XRD patterns, while for 240 °C, we observe an increase of refractive index of 0.1 even though there are no signs of crystallization in its relative XRD spectrum. We can assign this increase to the removal of organic content and residual solvent present in the sol, already highlighted in previous experiments.^34^ Although we observed the formation of the crystalline phase at 300 °C, no significant change in the refractive index compared to 240 °C is observed, which we assign to a small crystalline content. At 500 °C, where the crystallinity is evidently higher in comparison with 300 °C, we detect an increase of 0.17 in refractive index. Figure 1f also reports the Abbe number as a function of the annealing temperature. As the temperature increases, the value decreases from 15.9 to 13.6, in agreement with the formation of a dispersive anatase phase.^41^

**Figure 2:**
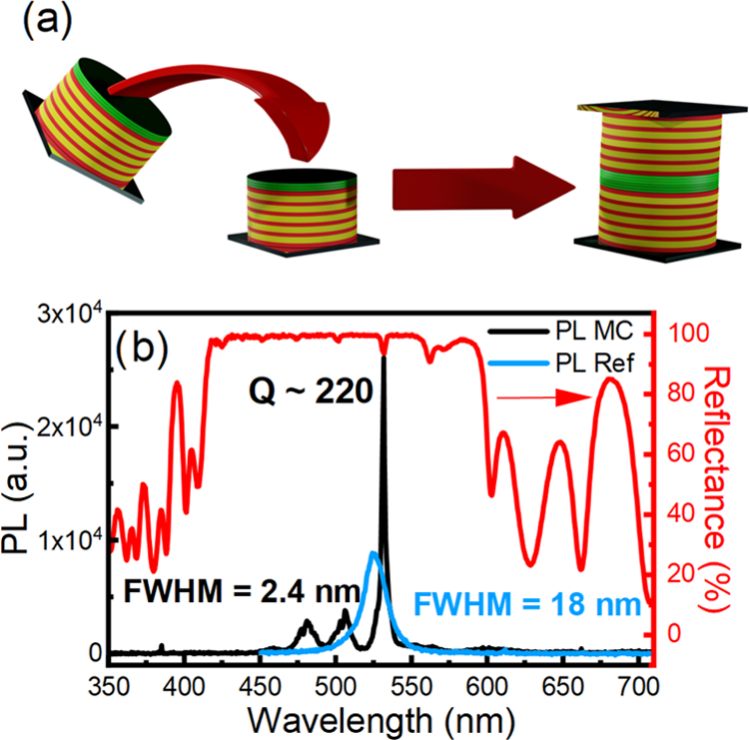
(a) Scheme of the MC fabrication process; (b) reflectance (red line) of MC and photoluminescence of perovskite nanocrystals both cast on glass (blue line) and embedded in the MC (black line).

After optimizing the DBR growth and materials treatment, we focused our attention on the deposition of the emitting medium in the cavity layer. Preparation of the CsPbBr_3_ NC film stack is carried out via a layer-by-layer (LbL) technique (adapted from ref (45)) via spin coating. After the depostion of every layer of CsPbBr_3_,^45^ a few microliters of methyl acetate, which is a known antisolvent for CsPbBr_3_ NCs, are dropped under dynamic conditions before depositing the subsequent layer in the same way. This step positively affects both the film formation and the optical properties by stripping original ligand molecules (oleate and oleylammonium) from the top of the film, thus preventing washout of emitting material during the subsequent deposition process.^46,47^ The construction of such a multilayer allows us to cast large amounts of emitters, which are highly sought for their applicability in various fields where it is often required for the production of a great amount of light such as lasing,^22,48^ while preserving the high-end optical properties typical of colloidal perovskite NCs. Moreover, working with highly reflective dielectric mirrors, we designed the cavity mode to maximize light production, therefore favoring its extraction from the closed microcavity system. Optical absorption and photoluminescence (PL) spectra for a 5-layer perovskite NC stack and its solution are shown in Figure S2 for comparison. The resulting multilayer preserves the optical quality of the supporting dielectric mirror with surface roughness below the nanometer (σ_RMS_ = 0.4 nm) as verified via atomic force microscopy (Figure S3). To form a microcavity, CsPbBr_3_ NC stacks were cast on two identical different DBRs with PBG tuned over the NC emission peak. The DBR was then pressed together and mechanically held in place to form the microcavity as shown in Figure 2a. Importantly, the obtained MC is considerably large (2.5 cm × 2.5 cm); thus, we expect a local variation of its spectral features.

Figure 2b shows the normal incidence reflectance and PL spectra of the MC compared to two stacks of bare NCs cast on glass substrates and then pressed together to replicate the microcavity layer. Both measurements on the microcavity are recorded on the same area. The reflectance data (red line) show a flat high-intensity signal extending from 420 to 600 nm assigned to the PBG. This band shows some narrow minima at 564, 533, 501, 475, 451, 440, and 426 nm, which are assigned to cavity modes, arising from the thick defect layer. The sample shows a slightly different response throughout its surface area, which is assigned to the different microcavity layer thickness achieved (Figure S4). The microcavity strongly affects the NC emission spectrum; in fact, the latter is suppressed in the PBG spectral region. The redistribution of the PL oscillator strength in the spectral window of the cavity mode is indeed responsible for a 3-fold increase in the PL intensity, while the full-width-at-half-maximum (fwhm) is reduced by 7.5 times, with the emission peak of the NCs in the MC being 2.4 nm in width. The MC quality factor (*Q*), (*Q* = λ_MAX_/Δλ_fwhm_) approaches 220, with an estimated finesse (*F*) ≈ 10 (*F* = Δω/κ, where Δω is the free spectral range corresponding to the optical mode spacing and κ is the cavity line width). To the best of our knowledge, polymer-based MCs show a *Q*-factor that spans from 80 to 150 for lasing applications^22,49^ to 255, which is, to date, the highest reported for specifically designed solution-processed microresonators, while a few reports of optical microcavities fabricated with sol–gel techniques share *Q* factors ranging between 20 and 50.^50,51^ Therefore, the value we report represents a record number for solution-processed systems comparable with state-of-the-art polymer MCs and unmatched by other solution-processed inorganic systems.

**Figure 3:**
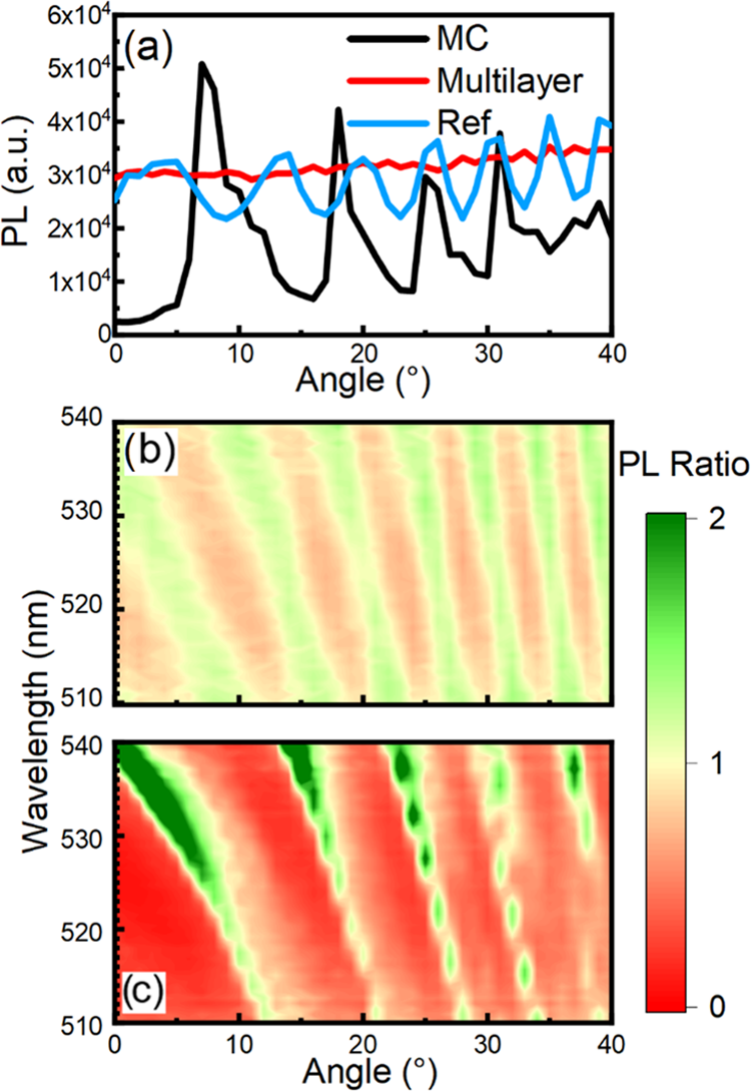
(a) Angle-resolved photoluminescence at a fixed wavelength (524 nm peak) for the “bare emitter” (red line), “MC reference” (blue line), and microcavity (black line); (b, c) photoluminescence ratio spectra with respect to the “bare emitter” for “MC reference” (panel b) and microcavity (panel c).

To highlight the directional photoluminescence (PL) enhancement effect, Figure 3a shows the PL intensity collected at 524 nm, corresponding to the maximum intensity for the bare nanocrystals as a function of the collection angle. The emission was excited with a 405 nm continuous wavelength laser. The data was compared to those of two references. The first reference (“bare emitter”) consists of two NC multilayers of comparable thickness cast on glass and sandwiched, while the second reference (“MC reference”) was fabricated to account for the structure outcoupling and consisted of only two titania layers cast on glass substrates enclosing the NCs, as in the MC sample. Schematics for all three analyzed samples and relative reflectance spectra are reported in Figure S5. Since both our sample and references present strong reflective optical features superimposing the laser excitation wavelength, we corrected the intensity of emission for the different excitation powers reaching the actual active layer. An extensive explanation of our approach can be found in the Supporting Information (Figures S6–S11). Therefore, this analysis represents a rough approximation and should be taken qualitatively.

For the “bare emitter” (red line, Figure 3a), we observe an almost constant intensity with a slight increase for higher collection angles, which we attribute to an increase in the amount of emitter excited when tilting the sample. For the “MC reference” (blue line, Figure 3a; here we mimic the PL outcoupling provided by the first and last interfaces of the DBRs sandwiching the emitter), we observe an oscillatory behavior of the PL intensity with the angle of collection due to the presence of an interference pattern able to modulate the PL. This pattern can be spectrally superimposed with the maxima and minima of the transmittance (Figure S10). However, as expected, the PL maximum for the “MC reference” shows an intensity comparable to that of the “bare emitter”. As for the PL of the emitter embedded in the MC (black line, Figure 3a), we found a similar behavior compared to the “MC reference” but with a different spacing between the oscillations. Furthermore, when the cavity modes spectrally overlap the native emission maximum of NCs, the PL intensity shows a 70% enhancement between 5 and 10°, while for higher angles, it is suppressed with few exceptions between 17−19° and 30−31°; these are due to the superposition of PL with the cavity modes (previously described and occurring at 564, 533, 501, 475, 451, 440, and 426 nm at normal incidence). This analysis highlights the effect on the PL at its native wavelength but does not consider the spectral redistribution of the emission that can be obtained inside the MC. In Figure 3b,c, we compare the PL of the “MC reference” and the MC by dividing their respective spectra by the spectrum of the “bare emitter” for all angles. The ratio spectra are shown as contour plots, where values above 1 (green tones) indicate PL enhancement, while values below 1 (red tones) indicate emission suppression. In the case of the “MC reference” (Figure 3b), we observe a periodic oscillatory behavior consistent with the angular dispersion of its transmittance spectrum (Figure S10). Such behavior is due to the interference pattern mentioned above. In addition, as expected, the soft color indicates subtle intensity changes compared to the “bare emitter” due to the absence of the photonic structure. For the emitter embedded in the cavity (Figure 3c), we instead observe stronger colors for both the enhancement and suppression regions. Starting from collection along the normal direction, the PL is redistributed toward longer wavelengths and gradually blue-shifted with an increasing angle, following the dispersion of the PBG and consequently of all spectral features. Again, comparing with panel a, we get the strongest enhancement effects in the region from normal to about 10° collection angle. These results confirm the possibility to obtain high dielectric contrast microcavities with a fully solution-based processing.

## Conclusions

In conclusion, we demonstrated a simple method to fabricate high-quality factor optical microcavities with inorganic materials entirely processed from solution. We reported the highest refractive index for solution-processed thin films of optical quality (*n*_sTiO_2__ = 2.17 at 550 nm), which allows us to fabricate DBRs and microcavities with a remarkable dielectric contrast of 0.73, coupling it with solution-processed silica. Exploiting these materials with perovskite nanocrystals, despite the well-known challenging manipulation of the latter when in solid state, allowed emitting cavities with a quality factor of 220, which is comparable with state-of-the-art solution-processed microresonators and unmatched by similar sol–gel systems. Moreover, we were also able to demonstrate between 70 and 300% of enhancement of the PL intensity for CsPbBr_3_ nanocrystals. These results suggest that, if properly designed, solution-processed microcavity can be used to arbitrarily both enhance and suppress light emission inside the cavity and open a new perspective in solution processing of functional inorganic cavities for a variety of applications such as, for example, lasing and improving the outcoupling of light in LEDs.

## Experimental Methods

### Precursor Solutions and Fabrication of the DBRs

Titanium(IV) butoxide (TIBU, 97% reagent grade), tetraethyl orthosilicate (TEOS, 98% reagent grade), poly(acrylic acid) (PAA) (*M*_w_ = 1800), 1-butanol(anhydrous), and hydrochloric acid (37% v/v) for the preparation of precursor solutions were purchased from Sigma-Aldrich. The titania precursor sol is prepared by adding a solution of TIBU in butanol (1.2 mol/L) to a butanol solution containing PAA (1 mg/mL) and a catalytic amount of HCl (100 μL) under stirring. The silica precursor sol is instead prepared by adding a solution of TEOS in butanol (2.1 mol/L) to a butanol solution containing PAA (33 mg/mL) and HCl (0.5 mL) under stirring. The solutions are kept under stirring for at least 4 h before usage. DBRs were fabricated by alternative dynamic spin coating of the two sols at 11,000 rounds per minute with short annealing processes (60 s) in between film deposition at 300 and 500 °C for sSiO_2_ and sTiO_2_, respectively.

### Synthesis of Nanocrystals and Fabrication of the PNC Multilayer

Lead bromide (PbBr_2_, 99,99%), cesium carbonate (Cs_2_CO_3_, reagent Plus, 99%), ethyl acetate (98.8%), methyl acetate (98.8%), toluene (anhydrous, 99.5%), 1-octadecene (ODE, technical grade, 90%), oleylamine (OLAm, technical grade 70%), and oleic acid (OA, 90%) were purchased from Sigma-Aldrich. CsPbBr_3_ nanocrystals were synthesized following state-of-the-art hot-injection method with Cs-oleate as a cation source.^52^ Perovskite multilayers were fabricated by depositing subsequent films via spin coating at 1800 rounds per minute, employing a layer-by-layer procedure^45^ described by Cirignano et al. In between every layer, a few microliters of methyl acetate (20 μL) are drop-cast during the rotation of the substrate.

### Characterization Techniques

Reflectance spectra were captured with a homemade optical setup using a y-fiber probe coupled with an AvaSpec-ULS2048CL-EVO-RS mounting a 25 μm slit for detection and a Mikropack DH-2000 deuterium-tungsten halogen as a white light source. Angle-resolved transmittance and steady-state photoluminescence were measured using a homemade optical fiber setup mounted on a rotating goniometer equipped with a tungsten-halogen HL-200-LL from Ocean Optics as a white light source and a 405 nm CW Oxxius laser for excitation. Detectors used were an AvaSpec-Mini2048CL-VI25, an AvaSpec-ULS2048CL-EVO-RS, and an AvaSpec-ULS2048 × 64-EVO for transmittance and PL. The optical function of thin films was retrieved via spectroscopic ellipsometry using a VASE ellipsometer by J.A. Woollam Co. Inc. Incidence angles ranging from 55 to 75° were adopted. Data analysis was performed through the dedicated software WVASE32. A Nanosurf CoreAFM was used for surface micrographs with a resolution of 512 lines in an area of 10 μm × 10 μm. Absorption measurements were collected using a Cary 5000 UV–vis–NIR spectrophotometer. X-ray diffraction patterns were acquired through a Rigaku MiniFlex 600 diffractometer, operating at 45 kV and 40 mA, equipped with a Cu Kα ceramic X-ray tube and a PIXcel3D 2 Å ∼ 2 area detector. The patterns were collected in the range of 2θ 5–60° with a scanning step of 0.02° and a scanning speed of 20°/min. Measurements in temperature were performed using an Anton Paar benchtop heating stage BTS 500 in air and with a heating ramp of 80 °C/min.

## Supporting Information Available

Additional schematics and data on the microcavity fabrication, optical absorption, and photoluminescence spectra of the nanocrystals in solution and film, and reference sample discussion and properties (PDF)

## References

[ref1] Nayak P. K.; Mahesh S.; Snaith H. J.; Cahen D. Photovoltaic Solar Cell Technologies: Analysing the State of the Art. Nat. Rev. Mater. 2019, 4 (4), 269–285. 10.1038/s41578-019-0097-0.

[ref2] Ballif C.; Haug F. J.; Boccard M.; Verlinden P. J.; Hahn G. Status and Perspectives of Crystalline Silicon Photovoltaics in Research and Industry. Nat. Rev. Mater. 2022, 7, 597–616. 10.1038/s41578-022-00423-2.

[ref3] Melchionna M.; Fornasiero P. Updates on the Roadmap for Photocatalysis. ACS Catal. 2020, 10 (10), 5493–5501. 10.1021/acscatal.0c01204.

[ref4] Xu Y. J. Promises and Challenges in Photocatalysis. Front. Catal. 2021, 1, 708319 10.3389/fctls.2021.708319.

[ref5] Ibhaze A. E.; Orukpe P. E.; Edeko F. O. High-Capacity Data Rate System: Review of Visible Light Communications Technology. J. Electron. Sci. Technol. 2020, 18 (3), 100055 10.1016/j.jnlest.2020.100055.

[ref6] Rahman M. T.; Bakibillah A. S. M.; Parthiban R.; Bakaul M. Review of Advanced Techniques for Multigigabit Visible Light Communication. IET Optoelectron. 2020, 14, 359–373. 10.1049/iet-opt.2019.0120.

[ref7] Zheng Z.-g.; Li Y.; Bisoyi H. K.; Wang L.; Bunning T. J.; Li Q. Three-Dimensional Control of the Helical Axis of a Chiral Nematic Liquid Crystal by Light. Nature 2016, 531 (7594), 352–356. 10.1038/nature17141. 26950601

[ref8] Bisoyi H. K.; Li Q. Light-Driven Liquid Crystalline Materials: From Photo-Induced Phase Transitions and Property Modulations to Applications. Chem. Rev. 2016, 116 (24), 15089–15166. 10.1021/acs.chemrev.6b00415. 27936632

[ref9] Zola R. S.; Bisoyi H. K.; Wang H.; Urbas A. M.; Bunning T. J.; Li Q. Dynamic Control of Light Direction Enabled by Stimuli-Responsive Liquid Crystal Gratings. Adv. Mater. 2019, 31 (7), 1806172 10.1002/adma.201806172. 30570775

[ref10] Joannopoulos J. D.; Villeneuve P. R.; Fan S. Photonic Crystals: Putting a New Twist on Light. Nature 1997, 386 (6621), 143–149. 10.1038/386143a0.

[ref11] Yablonovitch E. Inhibited Spontaneous Emission in Solid-State Physics and Electronics. Phys. Rev. Lett. 1987, 58 (20), 2059–2062. 10.1103/PhysRevLett.58.2059. 10034639

[ref12] Megahd H.; Comoretto D.; Lova P. Planar Microcavities: Materials and Processing for Light Control. Opt. Mater.: X. 2022, 13, 100130 10.1016/j.omx.2021.100130.

[ref13] Frezza L.; Patrini M.; Liscidini M.; Comoretto D. Directional Enhancement of Spontaneous Emission in Polymer Flexible Microcavities. J. Phys. Chem. C 2011, 115 (40), 19939–19946. 10.1021/jp206105r.

[ref14] Lova P.; Megahd H.; Stagnaro P.; Alloisio M.; Patrini M.; Comoretto D. Strategies for Dielectric Contrast Enhancement in 1D Planar Polymeric Photonic Crystals. Appl. Sci. 2020, 10, 4122 10.3390/app10124122.

[ref15] Malvezzi A. M.; Cattaneo F.; Vecchi G.; Falasconi M.; Guizzetti G.; Andreani L. C.; Romanato F.; Businaro L.; Fabrizio E. Di.; Passaseo A.; Vittorio M. De. Second-Harmonic Generation in Reflection and Diffraction by a GaAs Photonic-Crystal Waveguide. J. Opt. Soc. Am. B 2002, 19 (9), 2122–2128. 10.1364/JOSAB.19.002122.

[ref16] Lin B. C.; Chen K. J.; Han H. V.; Lan Y. P.; Chiu C. H.; Lin C. C.; Shih M. H.; Lee P. T.; Kuo H. C. Advantages of Blue LEDs with Graded-Composition AlGaN/GaN Superlattice EBL. IEEE Photonics Technol. Lett. 2013, 25 (21), 2062–2065. 10.1109/LPT.2013.2281068.

[ref17] Leem J. W.; Guan X.-Y.; Yu J. S. Tunable Distributed Bragg Reflectors with Wide-Angle and Broadband High-Reflectivity Using Nanoporous/Dense Titanium Dioxide Film Stacks for Visible Wavelength Applications. Opt. Express 2014, 22 (15), 18519–18526. 10.1364/OE.22.018519. 25089471

[ref18] Do Y. R.; Kim Y. C.; Song Y. W.; Cho C. O.; Jeon H.; Lee Y. J.; Kim S. H.; Lee Y. H. Enhanced Light Extraction from Organic Light-Emitting Diodes with 2D SiO2/SiNx Photonic Crystals. Adv. Mater. 2003, 15 (14), 1214–1218. 10.1002/adma.200304857.

[ref19] Vainos N. A. Laser Growth and Processing of Photonic Devices; Woodhead, 2012.

[ref20] Dodabalapur A.; Rothberg L. J.; Miller T. M.; Kwock E. W. Microcavity Effects in Organic Semiconductors. Appl. Phys. Lett. 1994, 64 (19), 2486–2488. 10.1063/1.111606.

[ref21] Anni M.; Gigli G.; Cingolani R.; Patanè S.; Arena A.; Allegrini M. Organic μ Cavities Based on Thermally Evaporated TeOx-LiF Distributed Bragg Reflectors. Appl. Phys. Lett. 2001, 79 (9), 1381–1383. 10.1063/1.1398323.

[ref22] Canazza G.; Scotognella F.; Lanzani G.; De Silvestri S.; Zavelani-Rossi M.; Comoretto D. Lasing from All-Polymer Microcavities. Laser Phys. Lett. 2014, 11 (3), 035804 10.1088/1612-2011/11/3/035804.

[ref23] Bensaid M. O.; Miloua R.; Ghalouci L.; Godey F.; Soldera A. Multiscale Design and Optimization of Polymer-Based Photonic Crystals for Solar Shielding. Sol. Energy Mater. Sol. Cells 2017, 171, 166–179. 10.1016/j.solmat.2017.06.016.

[ref24] Bhagat S. D.; Chatterjee J.; Chen B.; Stiegman A. E. High Refractive Index Polymers Based on Thiol-Ene Cross-Linking Using Polarizable Inorganic/Organic Monomers. Macromolecules 2012, 45 (3), 1174–1181. 10.1021/ma202467a.

[ref25] Wei Q.; Zan X.; Qiu X.; Öktem G.; Sahre K.; Kiriy A.; Voit B. High Refractive Index Hyperbranched Polymers Prepared by Two Naphthalene-Bearing Monomers via Thiol-Yne Reaction. Macromol. Chem. Phys. 2016, 217 (17), 1977–1984. 10.1002/macp.201600276.

[ref26] Griebel J. J.; Namnabat S.; Kim E. T.; Himmelhuber R.; Moronta D. H.; Chung W. J.; Simmonds A. G.; Kim K. J.; Van Der Laan J.; Nguyen N. A.; Dereniak E. L.; MacKay M. E.; Char K.; Glass R. S.; Norwood R. A.; Pyun J. New Infrared Transmitting Material via Inverse Vulcanization of Elemental Sulfur to Prepare High Refractive Index Polymers. Adv. Mater. 2014, 26 (19), 3014–3018. 10.1002/adma.201305607. 24659231

[ref27] Megahd H.; Oldani C.; Radice S.; Lanfranchi A.; Patrini M.; Lova P.; Comoretto D. Aquivion–Poly(N-Vinylcarbazole) Holistic Flory–Huggins Photonic Vapor Sensors. Adv. Opt. Mater. 2021, 9 (5), 2002006 10.1002/adom.202002006.

[ref28] Zhang Q.; Goh E. S. M.; Beuerman R.; Judeh Z.; Chan-Park M. B.; Chen T.; Xu R. Development of Optically Transparent ZnS/Poly(Vinylpyrrolidone) Nanocomposite Films with High Refractive Indices and High Abbe Numbers. J. Appl. Polym. Sci. 2013, 129 (4), 1793–1798. 10.1002/app.38883.

[ref29] Paquet C.; Cyr P. W.; Kumacheva E.; Manners I. Polyferrocenes: Metallopolymers with Tunable and High Refractive Indices. Chem. Commun. 2004, 4 (2), 234–235. 10.1039/b311934c. 14737565

[ref30] Megahd H.; Lova P.; Sardar S.; D’andrea C.; Lanfranchi A.; Koszarna B.; Patrini M.; Gryko D. T.; Comoretto D. All-Polymer Microcavities for the Fluorescence Radiative Rate Modification of a Diketopyrrolopyrrole Derivative. ACS Omega 2022, 7, 15499 10.1021/acsomega.2c00167. 35571840 PMC9096937

[ref31] Bachevillier S.; Yuan H. K.; Strang A.; Levitsky A.; Frey G. L.; Hafner A.; Bradley D. D. C.; Stavrinou P. N.; Stingelin N. Fully Solution-Processed Photonic Structures from Inorganic/Organic Molecular Hybrid Materials and Commodity Polymers. Adv. Funct. Mater. 2019, 29 (21), 1808152 10.1002/adfm.201808152.

[ref32] Anderson L. E.; Kleine T. S.; Zhang Y.; Phan D. D.; Namnabat S.; LaVilla E. A.; Konopka K. M.; Ruiz Diaz L.; Manchester M. S.; Schwiegerling J.; Glass R. S.; Mackay M. E.; Char K.; Norwood R. A.; Pyun J. Chalcogenide Hybrid Inorganic/Organic Polymers: Ultrahigh Refractive Index Polymers for Infrared Imaging. ACS Macro Lett. 2017, 6 (5), 500–504. 10.1021/acsmacrolett.7b00225. 35610885

[ref33] Russo M.; Campoy-Quiles M.; Lacharmoise P.; Ferenczi T. A. M.; Garriga M.; Caseri W. R.; Stingelin N. One-Pot Synthesis of Polymer/Inorganic Hybrids: Toward Readily Accessible, Low-Loss, and Highly Tunable Refractive Index Materials and Patterns. J. Polym. Sci., Part B: Polym. Phys. 2012, 50 (1), 65–74. 10.1002/polb.22373.

[ref34] Bertucci S.; Megahd H.; Dodero A.; Fiorito S.; Di Stasio F.; Patrini M.; Comoretto D.; Lova P. Mild Sol–Gel Conditions and High Dielectric Contrast: A Facile Processing toward Large-Scale Hybrid Photonic Crystals for Sensing and Photocatalysis. ACS Appl. Mater. Interfaces 2022, 14 (17), 19806–19817. 10.1021/acsami.1c23653. 35443778 PMC9073830

[ref35] D’Innocenzo V.; Srimath Kandada A. R.; De Bastiani M.; Gandini M.; Petrozza A. Tuning the Light Emission Properties by Band Gap Engineering in Hybrid Lead Halide Perovskite. J. Am. Chem. Soc. 2014, 136 (51), 17730–17733. 10.1021/ja511198f. 25469762

[ref36] Chung I.; Lee B.; He J.; Chang R. P. H.; Kanatzidis M. G. All-Solid-State Dye-Sensitized Solar Cells with High Efficiency. Nature 2012, 485 (7399), 486–489. 10.1038/nature11067. 22622574

[ref37] Kojima A.; Teshima K.; Shirai Y.; Miyasaka T. Organometal Halide Perovskites as Visible-Light Sensitizers for Photovoltaic Cells. J. Am. Chem. Soc. 2009, 131 (17), 6050–6051. 10.1021/ja809598r. 19366264

[ref38] Adhyaksa G. W. P.; Veldhuizen L. W.; Kuang Y.; Brittman S.; Schropp R. E. I.; Garnett E. C. Carrier Diffusion Lengths in Hybrid Perovskites: Processing, Composition, Aging, and Surface Passivation Effects. Chem. Mater. 2016, 28 (15), 5259–5263. 10.1021/acs.chemmater.6b00466.

[ref39] Swarnkar A.; Chulliyil R.; Ravi V. K.; Irfanullah M.; Chowdhury A.; Nag A. Colloidal CsPbBr3 Perovskite Nanocrystals: Luminescence beyond Traditional Quantum Dots. Angew. Chem., Int. Ed. 2015, 54 (51), 15424–15428. 10.1002/anie.201508276. 26546495

[ref40] Ahmed G. H.; Yin J.; Bakr O. M.; Mohammed O. F. Successes and Challenges of Core/Shell Lead Halide Perovskite Nanocrystals. ACS Energy Lett. 2021, 1340–1357. 10.1021/acsenergylett.1c00076.

[ref41] Jolivet A.; Labbé C.; Frilay C.; Debieu O.; Marie P.; Horcholle B.; Lemarié F.; Portier X.; Grygiel C.; Duprey S.; Jadwisienczak W.; Ingram D.; Upadhyay M.; David A.; Fouchet A.; Lüders U.; Cardin J. Structural, Optical, and Electrical Properties of TiO2 Thin Films Deposited by ALD: Impact of the Substrate, the Deposited Thickness and the Deposition Temperature. Appl. Surf. Sci. 2023, 608, 155214 10.1016/j.apsusc.2022.155214.

[ref42] Rodríguez-de Marcos L. V.; Larruquert J. I.; Méndez J. A.; Aznárez J. A. Self-Consistent Optical Constants of SiO2 and Ta2O5 Films. Opt. Mater. Express 2016, 6 (11), 3622–3637. 10.1364/OME.6.003622.

[ref43] Církva V.; Žabová H.; York N. Photocatalysis on Titania-Coated Electrode-Less Discharge Lamps; Nova Science Publisher, 2010, New York.

[ref44] Sharon M.; Modi F.; Sharon M. Titania Based Nanocomposites as a Photocatalyst: A Review. Mater. Sci. 2016, 3 (3), 1236 10.3934/matersci.2016.3.1236.

[ref45] Cirignano M.; Fiorito S.; Barelli M.; Aglieri V.; De Franco M.; Bahmani Jalali H.; Toma A.; Di Stasio F. Layer-by-Layer Assembly of CsPbX3 Nanocrystals into Large-Scale Homostructures. Nanoscale 2022, 14 (41), 15525–15532. 10.1039/D2NR04169C. 36239340 PMC9612634

[ref46] Swarnkar A.; Marshall A. R.; Sanehira E. M.; Chernomordik B. D.; Moore D. T.; Christians J. A.; Chakrabarti T.; Luther J. M. Quantum Dot–Induced Phase Stabilization of α-CsPbI3 Perovskite for High-Efficiency Photovoltaics. Science 2016, 354 (6308), 92–95. 10.1126/science.aag2700. 27846497

[ref47] Wheeler L. M.; Sanehira E. M.; Marshall A. R.; Schulz P.; Suri M.; Anderson N. C.; Christians J. A.; Nordlund D.; Sokaras D.; Kroll T.; Harvey S. P.; Berry J. J.; Lin L. Y.; Luther J. M. Targeted Ligand-Exchange Chemistry on Cesium Lead Halide Perovskite Quantum Dots for High-Efficiency Photovoltaics. J. Am. Chem. Soc. 2018, 140 (33), 10504–10513. 10.1021/jacs.8b04984. 30044630

[ref48] Michalzik R. VCSEL Fundamentals. In VCSELs: Fundamentals, Technology and Applications of Vertical-Cavity Surface-Emitting Lasers; Michalzik R., Ed.; Springer: Berlin Heidelberg, 2013; pp 19–75.

[ref49] Manfredi G.; Lova P.; Di Stasio F.; Krahne R.; Comoretto D. Directional Fluorescence Spectral Narrowing in All-Polymer Microcavities Doped with CdSe/CdS Dot-in-Rod Nanocrystals. ACS Photonics 2017, 4 (7), 1761–1769. 10.1021/acsphotonics.7b00330.

[ref50] Jasieniak J.; Sada C.; Chiasera A.; Ferrari M.; Martucci A.; Mulvaney P. Sol-Gel Based Vertical Optical Microcavities with Quantum Dot Defect Layers. Adv. Funct. Mater. 2008, 18 (23), 3772–3779. 10.1002/adfm.200800784.

[ref51] Almeida R. M.; Marques A. C.; Chiasera A.; Chiappini A.; Ferrari M. Rare-Earth Doped Photonic Crystal Microcavities Prepared by Sol-Gel. J. Non-Cryst. Solids 2007, 353 (5–7), 490–493. 10.1016/j.jnoncrysol.2006.10.015.

[ref52] Zheng W.; Wan Q.; Liu M.; Zhang Q.; Zhang C.; Yan R.; Feng X.; Kong L.; Li L. CsPbBr3 Nanocrystal Light-Emitting Diodes with Efficiency up to 13.4% Achieved by Careful Surface Engineering and Device Engineering. J. Phys. Chem. C 2021, 125 (5), 3110–3118. 10.1021/acs.jpcc.0c11085.

