# Supplementary material for: High Quality Factor in Solution-Processed Inorganic Microcavities Embedding CsPbBr3 Perovskite Nanocrystals
Source: ACS Appl Opt Mater. 2023 Jul 18;1(7):1343–9. doi: 10.1021/acsaom.3c00157 (PMC10391615; doi:10.1021/acsaom.3c00157)
Supplement: Supplementary file 1 — ot3c00157_si_001.pdf [file ot3c00157_si_001.pdf]

Supporting Information for

High Quality Factor in Solution-Processed Inorganic Microcavities embedding $CsPbBr_3$ Perovskite Nanocrystals

Simone Bertucci,[1,2] Andrea Escher,[2] Matilde Cirignano,[1,2] Manuela De Franco,[1,2] Federico Locardi,[2] Maddalena Patrini,[3] Davide Comoretto,[2] Paola Lova[2,*] and Francesco Di Stasio[1,*]

1 Photonic Nanomaterials, Istituto Italiano di Tecnologia, Via Morego 30, 16163 Genova, Italy

2 Dipartimento di Chimica e Chimica Industriale, Università degli Studi di Genova, Via Dodecaneso 31, 16146 Genova, Italy

3 Dipartimento di Fisica, Università degli Studi di Pavia, Via Agostino Bassi, 6, 27100, Pavia, Italy

Corresponding Author: francesco.distasio@iit.it, paola.lova@unige.it

CALCULATION OF ABBE NUMBERS:

Equation S1.

$$V_D = \frac{n_d - 1}{n_F - n_C}$$

Where n_c, n_d and n_F are the refractive indices at the Fraunhofer wavelengths, 656.3 nm, 587.56 nm and 486.1 nm respectively.

SCHEMATIC OF THE FILM DEPOSITION METHOD:

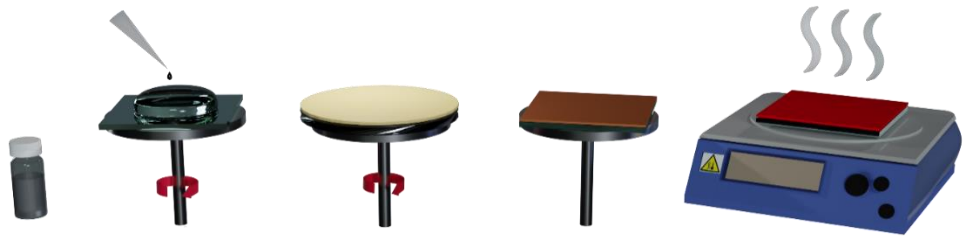

Figure S1. Schematic of the fabrication process from sol preparation to deposition, drying and annealing for a hybrid thin film.

OPTICAL ABSORPTION AND PHOTOLUMINESCENCE OF $CsPbBr_3$ NCs

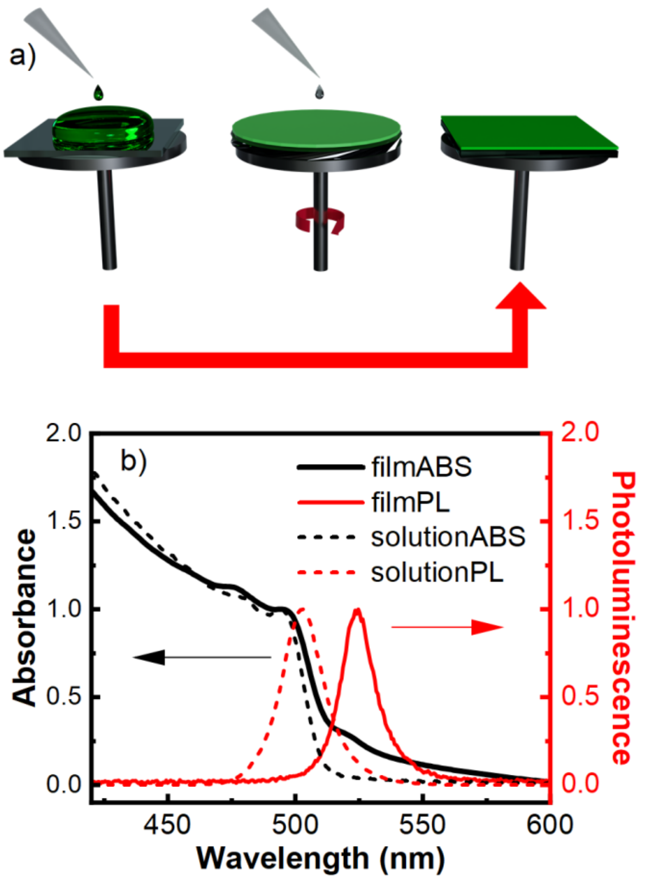

Figure S2. a) Schematic of NC multilayer deposition via LbL spin-casting; b) Normalized optical absorption and photoluminescence spectra for a 5-layers NC stack as compared to NC solution.

The perovskite nanocrystal (NC) film (solid black line) shows a structured and broad optical absorption with onset at about 510 nm similar to the solution absorption spectrum (dashed black line). The PL spectrum of the film (solid red line) consists in a sharp peak centered at 525 nm having a full width half maximum (FWHM) of 18 nm, while in solution (dashed red line) we observe a sharp peak centered at 503 with a FWHM of 19 nm. The PL red-shift is due to a change of dielectric medium in agreement with previous report in literature[1] and, we hypothesize the presence of a coalescence phenomenon passing from solution to solid state which can cause the mean average size of nanocrystals to increase. Such coalescence is suggested by the appearance of a secondary weak absorption peak at 519 nm in film. This phenomenon is well known for perovskite nanoplatelets[2] under irradiation. However, considering the two almost identical

FHWM, we observe no significant increase in polydispersity, thus signifying preservation of optical quality.

Furthermore, to confirm the optical quality of the system, we acquired AFM images on both the DBR and the DBR after the deposition of a nanocrystal multilayer, and calculated average surface roughness.

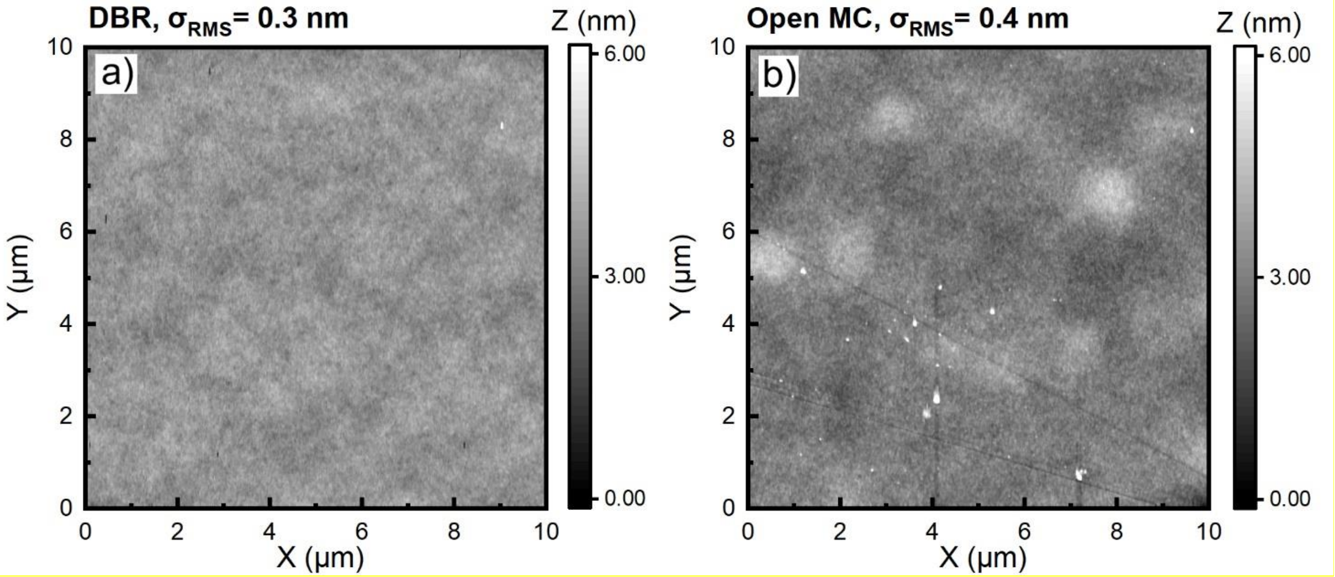

Figure S3. AFM images of a) DBR surface and b) NC multilayer surface on the DBR.

We can observe how the spin-coating process of multiple layers of nanocrystals does not impact the roughness of the system, thus preventing unwanted effects on the overall optical quality.

REFLECTANCE SPECTRA OF THE MICROCAVITY

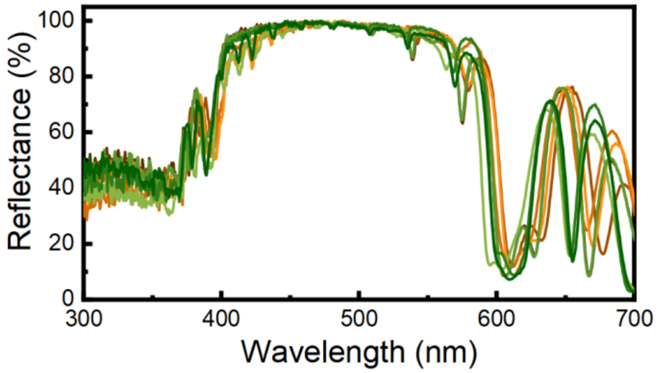

Figure S4. Different reflectance spectra collected on the MC surface area.

Reflectance spectra collected on different points of our microcavity (MC) sample. Slight spectral shifts in the optical features are attributed to the variability of cavity thickness intrinsic to the fabrication method we employed.

REFERENCE SAMPLES FOR THE CHARACTERIZATION OF THE MICROCAVITY

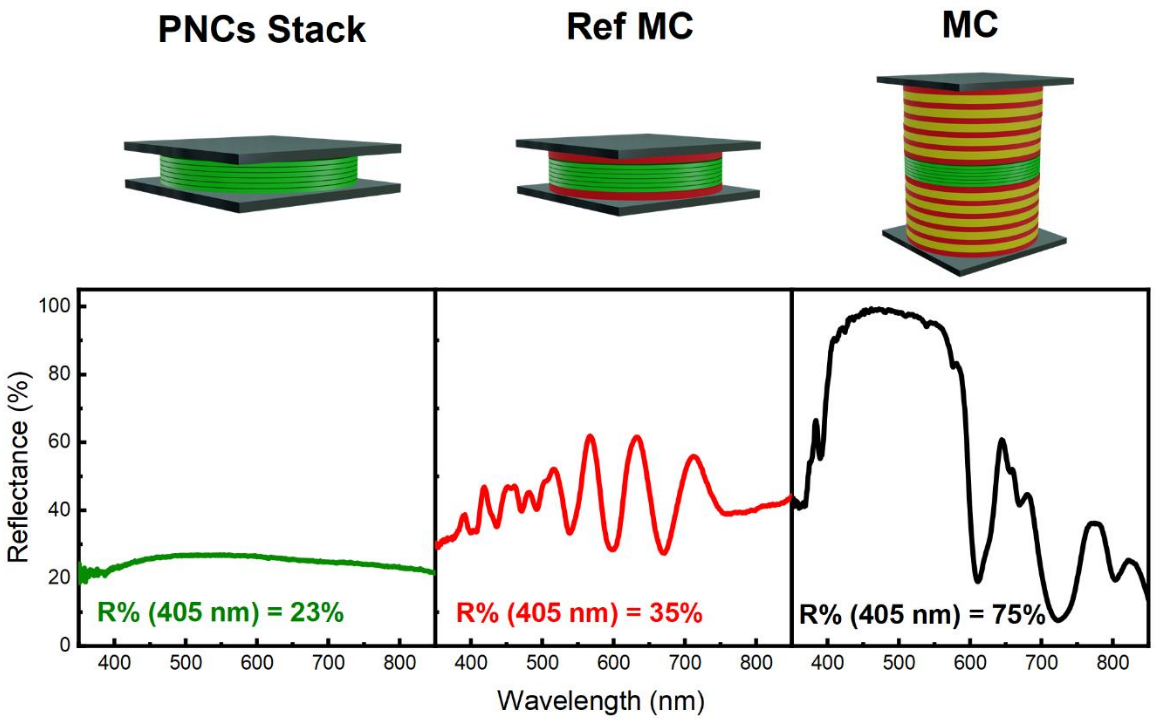

Figure S5. Schematic of MC and references with their relative reflectance spectra.

Reflectance spectra reported in Fig. S4 are averaged on multiple measurements carried on different points on the same sample, which gives rise to asymmetric features. The reflectance values collected at 405 nm are used for the PL intensity normalization (see main text Fig. 3).

To validate our assumption regarding photoluminescence intensity, as already mentioned in the paper, we must demonstrate:

1) Linear dependence between excitation power and intensity of PL emission for NCs.

2) Absence of non-negligible TiO_2 absorbance and light scattering phenomena at the pump wavelength

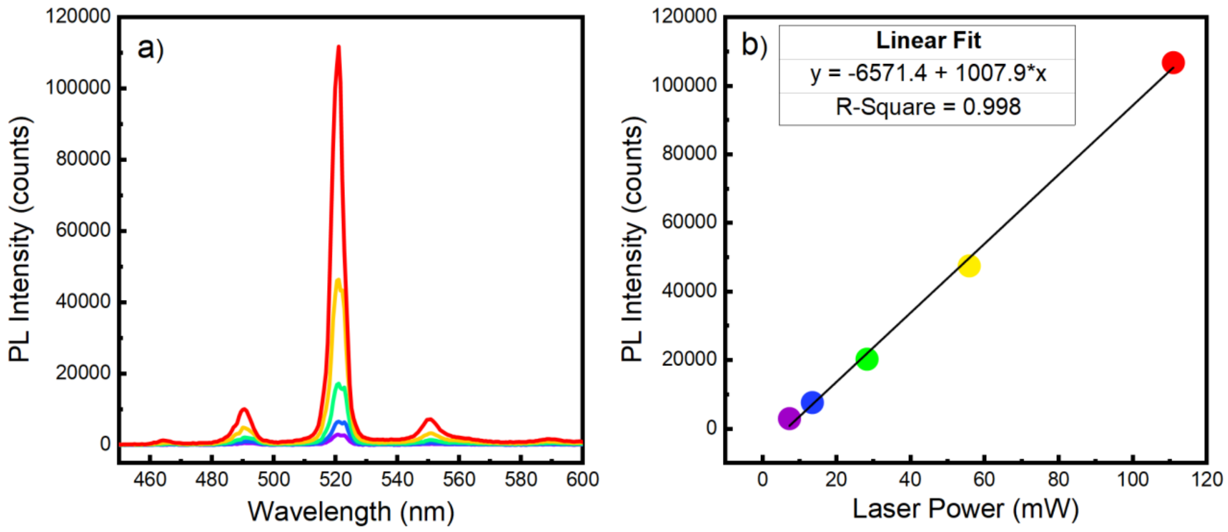

Figure S6. a) PL of the MC at increasing excitation power; b) maxima of emission intensity as a function of laser pump power, the data points were fit with a linear function.

In regards of (1), Figure S6a shows acquired PL spectra for a generic spot on the MC as a function of excitation laser power, while panel b shows the PL intensity maxima as a function of laser power. The intensity of emission is linearly dependent on the laser pump power. To highlight the linear behavior, we report the linear fit bearing R-Square value of 0.998, which validates our hypothesis. The data shown in Figure S6 were collected on a different area of the very same sample presented in the main text, hence the variation in spectral features.

We also report, in figure S7, the variation of PL peak linewidth with increasing power for the same spot analyzed in figure S6.

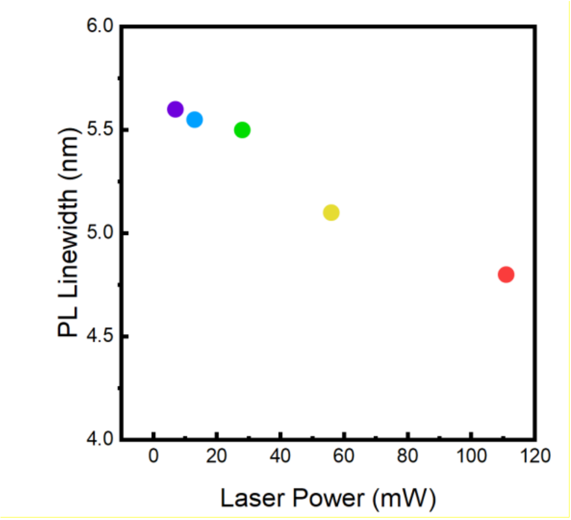

Figure S7. PL linewidth as a function of laser power.

We observe a slight decrease of about 1 nm in the PL peak linewidth which is comparable however with the resolution of our spectrometer.

In regards of (2), we measured the intensity of a laser beam at a fixed power. We then proceeded by shining the same beam through a DBR and collect again the signal from the laser. Finally, we compared the intensity loss with the reflectance of photonic structures superimposing the wavelength of the laser emission. Results are reported in Fig. S8.

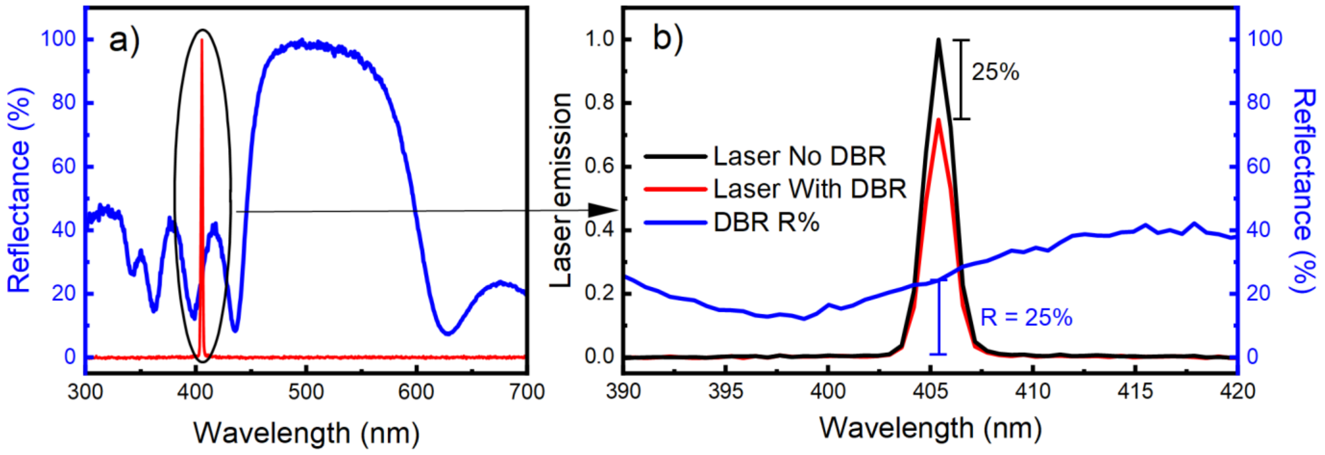

Figure S8. a) Reflectance spectrum for a 5.5 bilayers DBR and laser signal centered at 405 nm; b) close-up around the laser emission wavelength: normalized laser intensity without DBR (black line) and with DBR (red line) superimposing the reflectance spectrum of the DBR (blue line)

Fig. S8a panel shows the reflectance spectrum of a 5.5 bilayers titania-silica DBR tuned to the visible range with PBG spanning from 430 to 600 nm and laser signal acquired at 405. In panel b we magnify on the spectral range of interest where we can observe how interposing a DBR between laser and detection causes a decrease of intensity of 25% which is consistent with a 25% reflectance at 405 nm due to the interference fringes. This confirms the linear dependence between laser power attenuation and reflectance of photonic structure, and excludes major parasitic scattering and optical absorption phenomena, for instance from the TiO_2 films.

PHOTOLUMINESCENCE OF REFERENCES AND MICROCAVITY

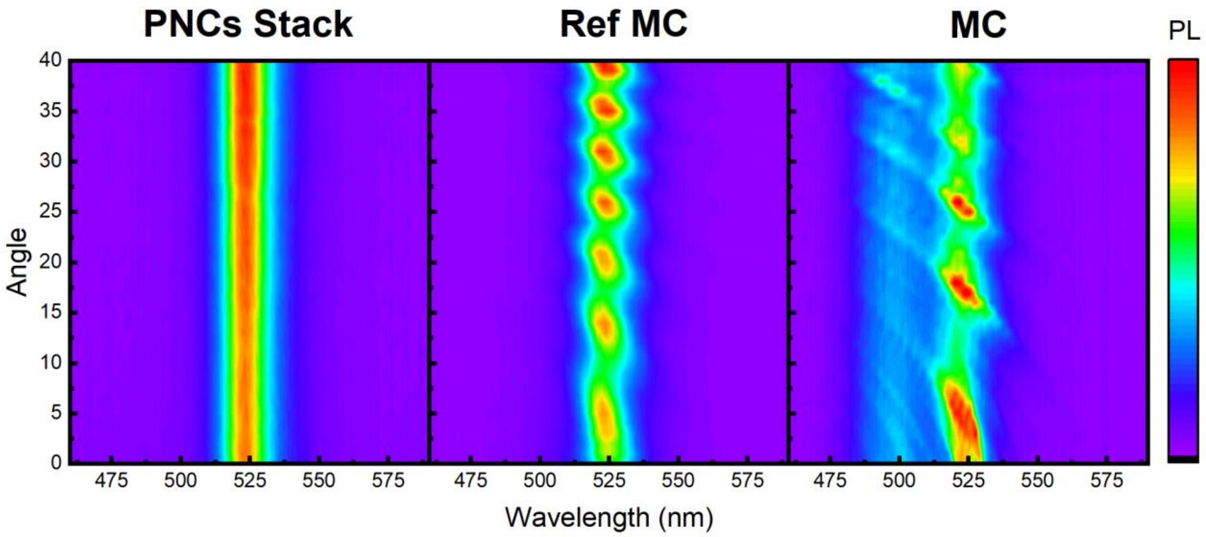

Figure S9. Angle resolved photoluminescence for references and MC samples.

Fig. S9 reports the PL dispersion between 0 and 40° for both the microcavity and the two reference samples which are used to plot the PL ratio contour plot in the main text (Fig. 3).

ANGLE RESOLVED TRANSMITTANCE SPECTRA

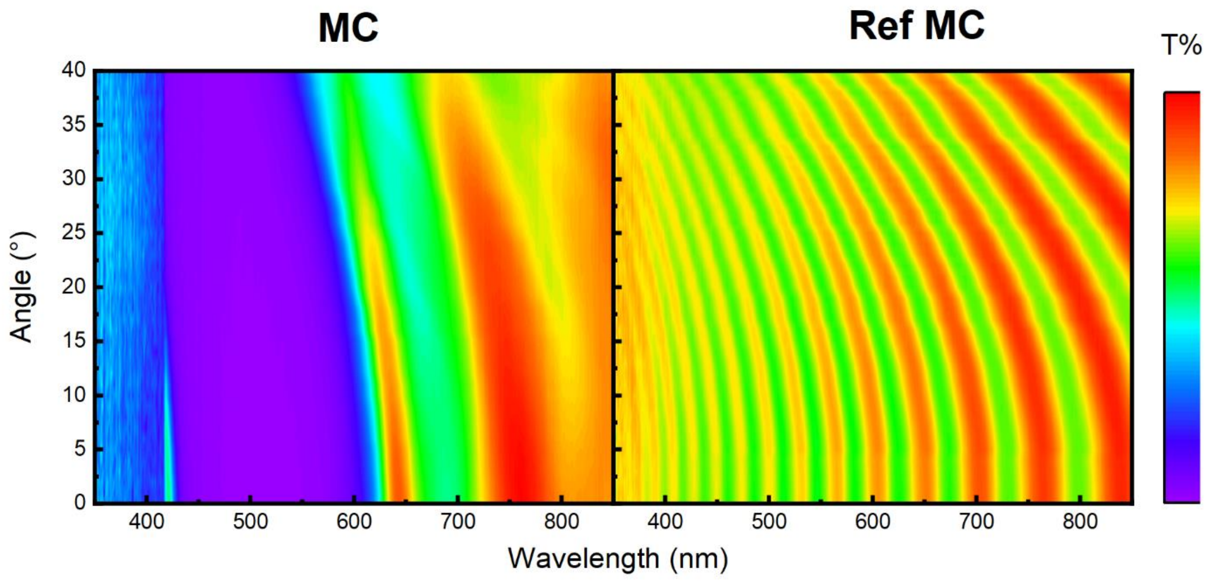

Figure S10. Angle resolved transmittance for the MC and the "Reference MC" sample.

Transmittance spectra here reported (Fig. S10) were collected for both samples in the same spot where the PL measurements used for Figure 3 were acquired. For the MC spectra, left panel, the signal is cut at 405 nm due to the presence of the long pass filter used for PL measurements. However, in the transmittance angular dispersion of the microcavity we do not observe optical features ascribable to cavity resonances due to lack of transmitted light for the specific system.

To this end, we acquired new transmission measurements using a high-sensitivity (i.e. high dynamic range) detector in the region of interest (Fig. S11).

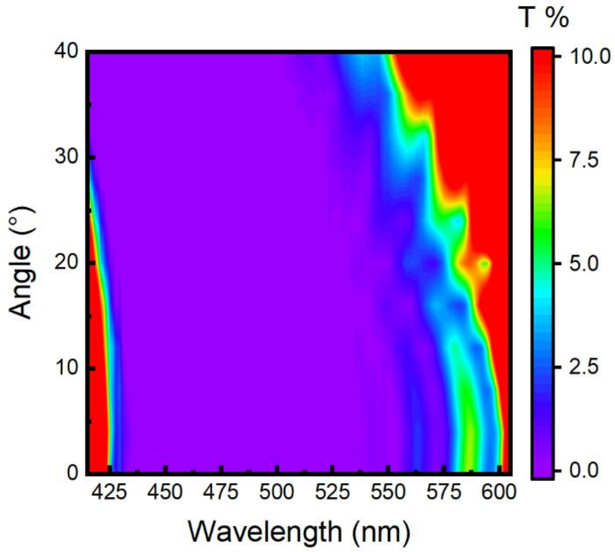

Figure S11. Angle resolved transmittance of the MC.

Although still being hardly detectable, we are able to observe additional spectral features within the MC. We can observe three different cavity resonances centered at 544, 563 and 586 nm with similar spacing compared to the ones visible in reflectance measurements. However, for our samples, reflectance is more suitable for a comprehensive description of the system.

References

(1) Baranov, D.; Toso, S.; Imran, M.; Manna, L. Investigation into the Photoluminescence Red Shift in Cesium Lead Bromide Nanocrystal Superlattices. *J. Phys. Chem. Lett.* **2019**, *10* (3), 655–660. https://doi.org/10.1021/acs.jpclett.9b00178.

(2) Shamsi, J.; Rastogi, P.; Caligiuri, V.; Abdelhady, A. L.; Spirito, D.; Manna, L.; Krahne, R. Bright-Emitting Perovskite Films by Large-Scale Synthesis and Photoinduced Solid-State Transformation of CsPbBr3 Nanoplatelets. *ACS Nano* **2017**, *11* (10), 10206–10213. https://doi.org/10.1021/acsnano.7b04761.
